# Draft Genome Sequences of Two “*Haemophilus quentini*” Isolates Recovered from Two Different Patients’ Blood Cultures

**DOI:** 10.1128/genomeA.01321-16

**Published:** 2016-11-23

**Authors:** Alireza Eshaghi, Deidre Soares, Raymond Tsang, David Richardson, Julianne V. Kus, Samir N. Patel

**Affiliations:** a Department of Clinical Laboratory and Microbiology Sciences, Public Health Laboratories, Public Health Ontario, Toronto, Ontario, Canada; b National Microbiology Laboratory, Public Health Agency of Canada, Winnipeg, Manitoba, Canada; c Departments of Medicine and Laboratory Medicine, William Osler Health System, Toronto, Ontario, Canada; d Department of Laboratory Medicine and Pathobiology, University of Toronto, Toronto, Ontario, Canada

## Abstract

Here, we present the draft genome sequences of two strains (K068 and C860) of the genospecies “*Haemophilus quentini*.” The isolates were recovered from blood cultures of a newborn neonate and an elderly patient with septicemia in Ontario, Canada.

## GENOME ANNOUNCEMENT

In humans, *Haemophilus influenzae* strains are often limited to the upper respiratory tract; however, there have been reports of infections in the urogenital tracts. Most of these urogenital infections are caused by nontypeable (NT) *H. influenzae* biotype IV strains (1, 2). Invasive cases of NT *H. influenzae* during the perinatal period have also been reported in the literature (3, 4). Molecular analysis of NT *H. influenzae* biotype IV identified a subset of closely related strains which were initially referred to as “*H. influenzae* cryptic genospecies,” or, “*Haemophilus quentini*” (5). *H. quentini* can only be clearly differentiated from *H. influenzae* using 16S rRNA full-gene sequencing, as traditional phenotypic and biochemical tests are not useful in identifying *H. quentini*. Interestingly, 16S rRNA gene sequence is more closely related to *Haemophilus haemolyticus* than *H. influenzae* (6). Here, we report draft genome sequences of two *H. quentini* isolates recovered from blood cultures from two different patients. The biochemical results suggested *H. influenzae*. However, it was ornithine positive. As a result, 16S rRNA sequencing was performed, which suggested *H. haemolyticus*. Due to the discrepant results, the cultures were sent to National Microbiology Laboratory (NML) for *H. quentini*-specific testing. The NML performed *H. quentini*-specific molecular testing to confirm their identification.

Genomic DNA of *H. quentini* isolates K068 and C860 was extracted and purified using a QIAamp DNA minikit (Qiagen, Valencia, CA, USA) from an overnight culture on a blood agar plate. The samples were indexed during library preparation using the Nextera XT DNA sample preparation kit (Illumina, San Diego, CA, USA). The sequencing library was quantified using Qubit 2.0 (Invitrogen, Waltham, MA, USA), and concentration and quality were analyzed by Bioanalyzer (Agilent Technologies, Richardson, TX, USA). The pooled libraries were sequenced using MiSeq Illumina with the V2 kit (2 × 150 bp), according to the manufacturer’s instructions, generating 2,735,812 and 1,308,728 high-quality reads corresponding to 407,844,516 and 190,672,282 detected bases for *H. quentini* strains K068 and C860, respectively.

The raw Illumina reads were trimmed and assembled using the *de novo* assembler in CLC Genomics Workbench version 8.0.1 (CLC bio, Germantown, MD, USA). The number of contigs per assembly was 63 and 78 for strains K068 and C860, respectively. All contigs less than 500 bp were filtered, and the remaining 59 contigs (*N*_50_, 69,953 bp) (K068) and 71 contigs (*N*_50_, 66,205 bp) (C860) were used for annotation. Using the NCBI Prokaryotic Genome Annotation Pipeline (PGAP) (http://www.ncbi.nlm.nih.gov/genome/annotation_prok), 1,989 predicted protein-coding sequences (CDSs), 7 rRNAs, and 48 tRNAs were annotated for *H. quentini* strain K068, while 1,983 CDSs, 5 rRNAs, and 49 tRNAs were annotated for *H. quentini* strain C860.

The functional comparison of the genome sequences available on the Rapid Annotations using Subsystems Technology (RAST) server revealed the closest neighbor of our *H. quentini* isolates to be *H. influenzae* R2866 (score, 507), followed by *H. influenzae* PittGG (score, 500) (7).

### Accession number(s).

The draft genome sequences for both isolates have been deposited at GenBank under the accession numbers MDJB00000000 (K068) and MDJC00000000 (C860).
